# Who Bites Me? A Tentative Discriminative Key to Diagnose Hematophagous Ectoparasites Biting Using Clinical Manifestations

**DOI:** 10.3390/diagnostics10050308

**Published:** 2020-05-15

**Authors:** Mohammad Akhoundi, Denis Sereno, Anthony Marteau, Christiane Bruel, Arezki Izri

**Affiliations:** 1 Parasitology-Mycology Department, Avicenne Hospital, AP-HP, 93000 Bobigny, France; anthony.marteau@aphp.fr (A.M.); arezki.izri@aphp.fr (A.I.); 2 MIVEGEC, IRD, Montpellier University, 34032 Montpellier, France; denis.sereno@ird.fr; 3 InterTryp, IRD, Montpellier University, 34032 Montpellier, France; 4 Agence Régionale de Santé (ARS) Île-de-France, 35, rue de la Gare, 75935 Paris CEDEX 19, France; christiane.bruel@ars.sante.fr

**Keywords:** hematophagous arthropods, blood feeding, bite spot, clinical manifestation, diagnosis

## Abstract

Arthropod blood feeders are vectors of several human pathogenic agents, including viruses (e.g., yellow fever, chikungunya, dengue fever), parasites (e.g., malaria, leishmaniasis, lymphatic filariasis), or bacteria (e.g., plague). Besides their role as a vector of pathogens, their biting activities cause a nuisance to humans. Herein, we document clinical symptoms associated with the biting of ten clusters of hematophagous arthropods, including mosquitoes, biting midges and sandflies, lice, ticks, tsetse flies, blackflies, horse flies, fleas, triatomine and bed bugs. Within the framework of clinical history and entomo-epidemiological information, we propose a tentative discriminative key that can be helpful for practicing physicians in identifying hematophagous arthropods biting humans and delivering treatment for the associated clinical disorders.

## 1. Introduction

Arthropods are a large group of invertebrates, having a significant impact on human health. They emerged in the late Precambrian period, approximately 550 million years ago (MYA) [1]. Bloodsucking arthropods are involved in the transmission of a wide diversity of pathogens such as bacteria, viruses, protozoa, and microfilariae, and are also a significant cause of nuisances for humans, worldwide (Table 1).

Arthropods biting occur for diverse purposes of defense mechanism, paralyzing, parasitizing their hosts, or for feeding [2]. Hematophagy has emerged in many orders and families, among arthropods like Anoplura (lice), Siphonaptera (fleas), Ixodida (ticks), Hemiptera (bed bugs and triatomines) and Diptera (mosquitoes) [3]. Blood feeder arthropods took the high nutritional value of blood for their own advantages. The feeding behavior is classified into two categories, explaining in part differences in clinical presentation and dermatological reactions: (i) telmatophagy/telmophagy (pool feeding), in which the feeders cut the epidermis and create a pool of blood that they suck (e.g., sandfly), or (ii) solenophagy (vessel feeding), in which the feeders insert their specialized mouthparts into the blood vessel for taking blood (e.g., mosquitoes and bed bugs) [4]. This feeding habit signifies them as temporary (e.g., mosquitoes, sandflies, tsetse flies or tabanids), permanent (e.g., lice) or periodic (e.g., fleas and ticks) ectoparasites [3]. It can be restricted to one sex (e.g., only female mosquitoes are blood feeder) or to a peculiar developmental stage (e.g., nymphal stage in bed bugs), or to both sexes and at all developmental life stages (e.g., lice, bed bugs).

Several protein-based factors present in the saliva facilitate blood-feeding. These include vasodilator and inhibitor agents of blood coagulation and platelet aggregation, as well as the proteins with the anesthetic role [2,5]. In general, reactions following arthropod bites are not specific and vary widely from one individual to another. The immunoglobulin E (IgE) and G (IgG) responses directed against these proteins of insect saliva are in part responsible for the individualized manifestation of arthropod bite [6]. Besides immunological reactions, several factors play a role in clinical manifestations. They include feeding behavior (solenophagy/telmatophagy), quietness, volume, and number (one or multiple bites by the same insect) of arthropod blood-feeding activity, but also the environmental temperature or host-associated cues (CO_2_ and heat), etc. [5]. The impact of vector-borne pathogens on the bite spot clinical manifestation is excluded in the present review.

## 2. Clinical Manifestations of Arthropod Bites

### 2.1. Culicidae (Mosquitoes)

**Figure 1:**
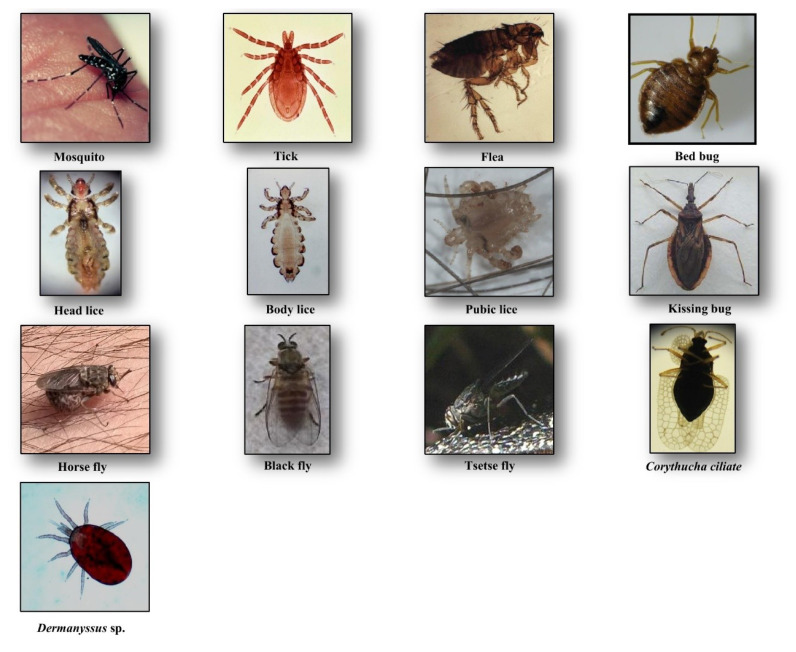
Hematophagous arthropods feeding on human blood.

Culicidae belongs to the Diptera order and Nematocera sub-order. To date, 3546 species from 111 genera are described worldwide, which small numbers of them feed on humans [7]. They are the largest vector group of pathogens, transmissible to humans (i.e., *Plasmodium* sp., filariae, and arboviruses). These vessel feeders (solenophagy) possess long mouthparts for piercing and blood-sucking with a feeding habit restricted to females (Figure 1). In general, mosquito bites occur during sunset or at night, but some species may bite daytime (e.g., *Aedes* sp.) [8].

Clinical manifestations: Reactions to mosquitos’ initial bite varies in severity between individuals, and delayed local skin reactions could appear after a second exposition. After repeated bites, pruritic papules develop quickly on the skin. People experiencing continuous exposition of the same mosquito species could encounter a loss of the immediate reaction towards biting. Some people may express more adverse serious reactions, like blistering or large skin rash, accompanied by fever [9]. The hypersensitivity to mosquito bites (HMB) is characterized by an intense local skin reaction with fever and regional lymphadenopathy [10]. This affection is mainly reported in south-eastern Asian countries. In the absence of immediate care, anaphylactic shock may occur, which can be fatal.

**Figure 2:**
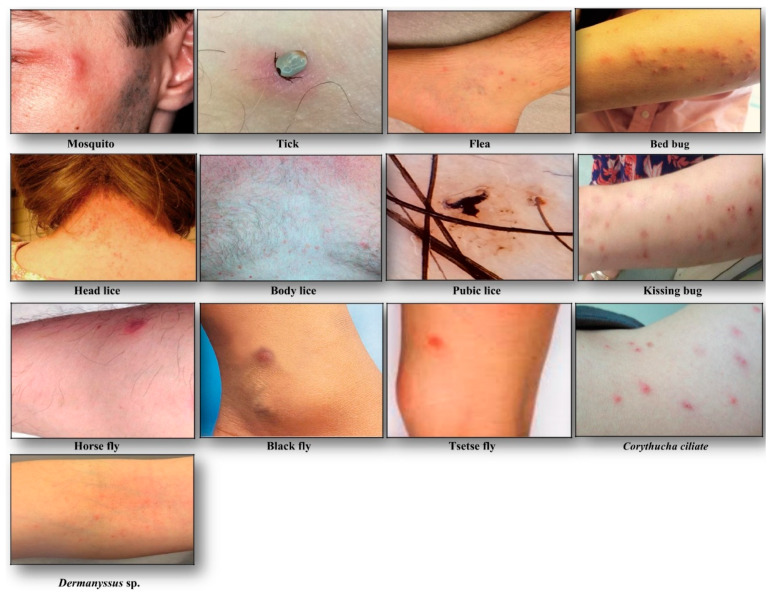
Clinical manifestations following hematophagous arthropods biting.

Bite spot diagnosis: Bites appear most commonly in the exposed body area, they are individualized and scattered on the skin (see Figure 2). The common cutaneous manifestations to mosquito bites consist typically of red itchy papules that resolve within few hours to several days [11]. The biting activity is strongly season-dependent, occurring commonly during sunset and night in summer of temperate regions, and all the year in the tropical region.

### 2.2. Ceratopogonidae (Biting Midges) and Phlebotominae (Sandflies)

Ceratopogonidae or biting midges include more than 5000 species with a worldwide distribution [12]. They are small insects with a length of 1–3 mm. They are the vector of infectious diseases of veterinary importance, like bluetongue disease, mansonellosis, African horse sickness, and epizootic hemorrhagic disease [13]. Members of the Phlebotominae family (sand flies) are insects of about 2–4 mm in length, holding their wings in vertical V-shape during resting time. They are proven vectors of viral and non-viral diseases, including bartonellosis, arboviruses and leishmaniasis caused by *Bartonella* sp., *Phlebovirus* or *Vesiculovirus* and *Leishmania* sp., respectively [14]. The female members of both families are pool feeders (telmophagy) (Table 1).

Clinical manifestation: Biting midges can cause acute discomfort, irritation, and severe local reactions. The latter is characterized by an acute pruritus, eczema or hypersensitivity. In the case of repeated biting, people may become desensitized, expressing a mild or no reaction [15].

Bite spot diagnosis: Bites caused by “biting midges” are painful and itchy with clinical symptoms that range from small reddish bump and a burning sensation at the bite spot to local irritations that cause significant itching [16]. Sandfly bites are also painful and cause small red bumps and blisters, but often remain unnoticed. These bumps and blisters can become itchy, infected or cause dermatitis or skin inflammation, and can persist for days or weeks [17] (Table 2).

### 2.3. Pediculidae, Pthiridae (Lice)

Lice are blood-sucking insects, obligate ectoparasites, widely distributed around the world. They belong to the order of Phthiraptera Haeckel, 1896, which has about 5000 species, parasites of warm-blooded animals [18]. The Anoplura sub-order has more than 550 species; most of them express strict host specificity [19]. Only three taxa are obligate ectoparasites of humans and are of concern for public health, including head lice, body lice, and pubic lice (Figure 1).

Clinical manifestation and bite spot diagnosis: Biting occurs all year long with localization on the human body that depends on the species.

Head lice (*Pediculus humanus capitis* de Geer, 1778) are obligate ectoparasites of 2 to 3.5 mm long, widespread throughout the world particularly among school-aged children [20] (Figure 1). The infestation occurs in all socio-economical levels and all ethnic groups. Males, females and larvae are strict hematophagous and feed exclusively on human blood, several times a day and reside close to the scalp, in order to maintain its body temperature and humidity. The eggs (nits) are laid by females and cemented at the base of the hair using an “adhesive” secretion produced by the female called cementum [21]. They take advantage of the slightest direct contact, head to head, to grab the hair of the new host. This passage from one host to another during direct contact is the main mode of transmission. Common symptoms of head lice bites are intense pruritus and papules on the scalp, neck, ears, and shoulders [21]. The bites often appear as small reddish or pink bumps sometimes with coagulated blood (Figure 2). The rash caused by pruritic reaction and sleeping disturbance due to irritability, are among additional symptoms. The role of *Pediculus h. capitis* in disease transmission is debatable but in spite of detection of several pathogens in this insect, no formal evidence certifying its vectorial role is available [22].

Body lice (*Pediculus humanus humanus* Linnaeus, 1758) are morphologically similar to head lice (3 to 4 mm length) (Figure 1). They are common in populations, suffering from a lack of standard hygienic conditions, such as homeless, prisoners, and war refugees [23]. Up to now, only body lice are reported as the recognized vectors of at least three pathogens namely *Rickettsia prowazekii* (epidemic typhus), *Bartonella quintana* (trench fever), and *Borrelia recurrentis* (louse-borne relapsing fever) [24] (Table 1). Body lice can be found alive in the belongings of an infected person, and be transferred to another one by close contact or by the exchange of infested clothing. The symptoms of biting are pruritus and small red lesions at the biting spots or skin rash, which develops to papule. In the case of massive infestations, hematomas or melanodermic lesions are sometimes observed (Figure 2, Table 2). In the case of continuous scratching, the bite spots can be itchy and can lead to secondary bacterial or fungal infection. Bite spots are observed in various body parts, in particular, chest, armpits, and other hairy parts. In persistent infestation cases, the skin may become thickened and discolored (vagabond’s disease) [25]. In the case of an allergic reaction, pruritus and rash are common visible symptoms, observed throughout the body. The discriminating symptom caused by body lice with those caused by pubic lice is the severe itch and pruritus, observed in the case of pubic lice infestation [26].

Pubic lice (*Pthirus pubis* Linnaeus, 1758) are yellow-gray ectoparasites, smaller (1–2 mm in length) than the body and head lice (Figure 1). They are found in the pubic region, and the transmission occurs by close and/or sexual contact [27]. Nevertheless, they are also found in other body parts, like eyelashes, armpit, chest or facial hairs [27]. Itching in the genital region that becomes more intense at nights, because of lice activity, is a common sign of pubic lice infestation [28]. The itching is due to an allergic reaction to lice saliva. Scratching can cause additional inflammation and irritation with blue spots or small spots of blood on the skin [27] (Figure 2). Other symptoms include occasional pruritus, fever, irritability, and bluish spots near the bite spots.

### 2.4. Ixodidae, Argasidae (Ticks)

Ticks are blood-sucking ectoparasites of mammals, birds, and reptiles (Figure 1). About 850 species of tick are described [29]. Although they are active throughout the year, most of the tick bites occur in spring and summer. Life cycle of the ticks includes a six-legged larval stage and one or more eight-legged nymphal stages. These immature stages need a blood-meal before proceeding to the next stage. Two morphological and biological distinct families of ticks are described, Ixodidae (hard ticks) comprising about 700 species and Argasidae (soft ticks) comprising about 200 species [30]. The mouthparts in hard ticks are visible at the front of the body, differentiating them from the soft ticks [31]. Hard ticks take one day to one week to resume feeding, while soft ticks feed quickly and leave their host. They are vectors of a wide range of pathogens, including bacteria, rickettsia, protozoa, and viruses, causing Lyme disease, tick-borne encephalitis, Crimean–Congo hemorrhagic fever, rocky mountain spotted fever, tularemia, Colorado tick fever, human tick-borne ehrlichiosis, babesiosis, tick paralysis, relapsing fever, and the Mediterranean spotted fever [31] (Table 1).

Clinical manifestation: Ticks cause acute or chronic skin diseases via physical trauma, salivary secretions, feces or body part deposition [32]. When a tick attaches to his host, the initial lesion development is due to the toxicity and the anti-coagulating activity of tick saliva, but also caused by the physical injuries caused by mouthparts of the tick. Tick bites can cause a flu-like symptom, vomiting, and even an anaphylactic shock [33]. They can also give symptoms by themselves with their delivered saliva toxin (paralysis tick). They prefer warm and moist areas of the body; therefore once they attach to their host, they migrate to other body parts, where they find adequate conditions to survive, like armpits or groins [34].

Bite spot diagnosis: Tick bites are most of the time painless. Bite spots are well separated and sporadic, occurring generally in uncovered body parts, most commonly lower limbs [35]. In the case of an attached tick, it can be removed and identified at the species level. The bite spot presentation is similar to a volcanic papule with a central hole (“black dot”), and reddish wheal or plaque (Figure 2, Table 2). The lesion can be hard, itchy or surrounded by redness. Skin lesions can persist and evolve to papules, nodules or necrotic black spots [35]. The latter is a scar with a crust. The acute pruritic papular dermatitis is a symptom, occurring following tick bites. Secondary infection of bite spots by bacteria can also be observed.

### 2.5. Glossinidae (Tsetse Flies)

Tsetse flies or *Glossina* spp. are hematophagous flies with about 6 to 15 mm in length (Figure 1), transmitting African trypanosomiasis (sleeping sickness), caused by *Trypanosoma brucei* (Kinetoplastida: Trypanosomatidae) in sub-Saharan Africa [36] (Table 1). About 30 species and subspecies of tsetse flies are known divided into three distinct groups or subgenera: *Austenia* (*G. fusca* group), *Nemorhina* (*G. palpalis* group) and *Glossina* (*G. morsitans* group) [37]. Both female and male tsetse flies feed exclusively on blood. The female is larviparous. Most tsetse flies are diurnal and active all the year. They are differentiated from other biting flies by their forward-pointing mouthparts (proboscis) and characteristic wing venation [38]. They are prevalent mainly in Central Africa. Besides their vectorial role, they cause African animal trypanosomiasis (nagana disease) resulting in gradual health decline in infected livestock, reduced milk and meat production, and increased abortion rates [39].

Clinical manifestation: The most common clinical signs of tsetse bites are reddish bumps or small red ulcers at the bite spot.

Bite spot diagnosis: The bite of tsetse fly is often painful and can develop a reddish sore (chancre) or itchy skin rash that may evolve after several days, to a boil-like swelling (Figure 2, Table 2).

### 2.6. Pulicidae (Fleas)

Fleas are small (1.5 to 4 mm in length), wingless and flat insects with three pairs of legs adapted for jumping (Figure 1). They feed exclusively on the blood of mammals and birds. About 2574 species belonging to 16 families and 238 genera have been described, but only a minority is synanthropic, that is they live in close association with humans [40,41]. They are vectors of bacterial (e.g., *Yersinia pestis*, *Bartonella henselae*, *Rickettsia typhi* and *R. felis*) and viral (e.g., Myxoma virus and feline leukemia virus) diseases [42] (Table 1). Among flea species, *Pulex irritans* favors blood-feeding on humans. The oriental rat flea, *Xenopsylla cheopis*, is the primary vector of *Yersinia pestis*, a Gram-negative coccobacillus, which causes plague [43]. People can be bitten by fleas via contact with an infected animal. Both sexes at the adult stage are hematophagous and bite at any time while active throughout the year [44]. The larvae feed mainly on organic debris left on their host’s skin or on the feces (dried blood) expelled by the adults which accumulates, along with the eggs.

Clinical manifestation: Flea bites are very itchy, with surrounding skin that may become sore or painful. They also cause skin irritation, flea bite allergy, hair loss, and reddish skin. Bites in humans are usually located in lower extremities like legs or ankles [45]. They can also cause anemia in extreme cases [46]. Persistent scratching can further damage the skin and cause a secondary bacterial infection.

Bite spot diagnosis: The most commonly observed symptom is small itchy red bumps, surrounded by reddish inflamed skin. The lesion initially exhibits punctuate hemorrhagic area representing the site of probing by the insect. Bites occur sporadically or in clusters and sometimes form a scatter or line pattern on the skin [45] (Figure 2, Table 2).

### 2.7. Cimicidae (Bed Bugs)

*Cimex lectularius* and *C. hemipterus,* commonly named “bed bugs”, are parasitic insects of 5 to 7 mm in length (Figure 1). They have a major impact on public health and are probably one of the most common ectoparasites with a worldwide distribution. Both sexes at all stages feed on a human. Infestation occurs in all ethnic groups and at all socioeconomic levels. During the two last decades, the infestation of human habitats has drastically increased, leading to rising concerns about bed bugs. They are responsible for several clinical and psychological disorders [47]. They are also suspected of being involved in the transmission of over 40 infectious agents. These include bacteria (e.g., *Borrelia recurrentis*, *B. duttoni*, *Coxiella burnetii* and *Rickettsia rickettsii*), fungi (e.g., *Aspergillus* spp.), viruses (e.g., Hepatitis B and HIV), filariae and parasites (e.g., *Trypanosoma cruzi*) [47] (Table 1). The competence of *C. lectularius* to act as a vector of *T. cruzi* (responsible agent of Chagas disease) and *Bartonella quintana* (causative agent of trench fever), has been recently probed in the laboratory [48,49], but no evidence on the vectorial role of *Cimex* spp. in the endemic areas is available [47].

Clinical manifestation: At least 10% to 30% of the people who are bitten do not develop any reactions [15]. Nevertheless, the bed bugs bites can cause a wide spectrum of dermatological manifestations that at least in part, depend on the individual’s immune response. These reactions include macule, papule, nodule, vesicle, bullae, erythematous lesion, pruritic reaction, secondary infection, and systemic sign [47]. The most commonly encountered clinical signs, following bed bug bites are pruritic maculopapular and erythematous lesions [47]. In the case of repeated bites, enlarged pruritus can occur. Symptoms often occur immediately, but in some cases, bullous rashes can emerge in the following few days. Without further irritation, symptoms typically resolve spontaneously after one to two weeks. After persistent scratching, a secondary bacterial or fungal infection may also occur. In rare cases, some other clinical disorders such as asthmatic reaction, urticaria or anaphylactic shock are observed [50].

Bite spot diagnosis: Bed bug bites are painless and occur on any exposed part of the body. Bed bugs feed mostly at night, but in case of hyper-infestation, their activity can take place all day long. No seasonality is noticed. Lesions are typically distributed in a linear or zigzag pattern, so-called “breakfast, lunch, dinner” bite pattern [47] (Figure 2, Table 2).

### 2.8. Reduviidae (Kissing Bugs)

Triatomine bugs are hematophagous insects with a long, cone-shaped head and a dark brown or black body (¾ to 1¼ inches) that are mostly active at night. They comprise 15 genera and 148 species and subspecies that are mainly found in Latin and South America, with a few species present in Asia, Africa, and Australia [51,52]. Triatomine bugs are a proven vector of *T. cruzi*, causative agent of American trypanosomiasis, called chagas disease (Figure 1). The bugs ingest the parasites when they feed on an infected animal or person. Infected bugs then deposit the parasites with their feces on the skin of another person during or shortly after feeding. Scratching or rubbing the parasites leads to enter the body through the bite wound or broken skin [53]. The disease is associated with poverty in rural areas or in favelas. Both sexes and all the five instars feed on blood (Table 1).

Clinical manifestation: The symptoms can be quite variable and range from no symptoms to severe and distressing signs. The common symptoms include skin rash, swollen lymph nodes, fever, headaches, body aches, fatigue, and nausea [54]. Proteins released via their bites can provoke anaphylaxis in sensitized individuals [55].

Bite spot diagnosis: Triatomine bugs’ bite occurs in any exposed body region, including the face, head, legs, and arms. They are generally painless and asymptomatic, but in some occasions can cause papules with hemorrhagic puncta, vesiculobullous lesions, eczematic reaction (the surrounding skin of bite spot might become red, swollen and itchy) or anaphylactic shock [56]. Swelling, redness of skin (called chagomas), and rash are common observable signs of kissing bugs’ bite (Figure 2, Table 2).

### 2.9. Simuliidae (Blackflies)

Simuliidae is a medically important family with over 2141 species, formally described [57]. They are usually small (1.5–4 mm), black, gray or brown, with short legs and antennae (Figure 1). Simulids are cosmopolitan insects, which are found in running water, near rivers, waterfalls, etc., where the larvae are fixed under stones or vegetable matter using the abdomen apex [58]. They are active in the morning and in the evening. Adult males feed on nectar, while females feed on blood before laying eggs. They are vectors of human diseases, notably human onchocerciasis or river blindness, the second ranking cause of infectious blindness [59]. The latter is caused by the nematode *Onchocerca volvulus*, and transmitted by *Simulium damnosum* (*S. damnosum*) and *S. neavei* in Africa and *S. callidum*, *S. metallicum* and *S. ochraceum* in Central and South America [60] (Table 1).

Clinical manifestation: “Black fly fever” is caused by the intense feeding of these flies. It is accompanied by headache, nausea, fever, and swollen lymph nodes. A severe anaphylactic reaction is a less common manifestation that may require hospitalization [61].

Bite spot diagnosis: Since females cut a small hole in the skin for blood-feeding (telmophagous), their biting spot becomes painful and itchy. Feeding is facilitated by anticoagulant proteins in the flies’ saliva that reduces the host’s awareness of being bitten, extending the feeding time. Various clinical presentations are described (edematous, erythematous-edematous, erysipeloid, inflammatory-indurative, phlegmonoid, and hemorrhagic), but the itching, localized swelling, inflammation eruptions of pruritic papules, vesicles, intense pruritus, and erythematous wheals are common symptoms, resulting from a hypersensitive reaction to black fly bites [62] (Figure 2, Table 2). The *Simulium* dermatitis is a concern with a high risk of anaphylaxis and acute cardiotoxicity in hypersensitive individuals [61].

### 2.10. Tabanidae (Horse Flies)

Horse-flies are a large family of the order Diptera with 4455 species belonging to 137 genera that mostly have a diurnal activity [63]. They are found all over the world, except in polar regions. Tabanid species have a body length between 5 and 25 mm mostly occur in warm areas with suitable moist locations for breeding [64]. Because of weak mouthparts, males do not feed on blood, but in contrast, the females bite animals to get enough protein for egg production. The mouthparts of females cut the skin to form a pool of blood, so they then aspirate (Figure 1). They are known to act as biological or mechanical vectors of various pathogens such as Loa loa worm, equine infectious anemia virus, *Trypanosoma* spp., cattle, and sheep anthrax and tularemia [65] (Table 1).

Clinical manifestation: The biting spot is painful due to the physical action of the fly’s mouthparts. Clinical manifestation includes large red and raised rash or temporary swollen skin. In exceptional cases, reactions to saliva may result in general toxicity and immune response that lead to stress and immunosuppression [66].

Bite spot diagnosis: The bite spots are usually red surrounded by a raised skin (Figure 2, Table 2). The pain, redness, and weal help to identify horsefly bites. Other symptoms may include urticaria, dizziness, weakness, wheezing, and angioedema (a temporary itchy, pink or red swelling occurring around the eyes or lips) [67].

## 3. Other Non-Common Hematophagous Arthropods

Identification of clinical manifestations due to these arthropods’ bites can be difficult for physicians who are not familiar with these arthropods, and the emerging associated clinical disorders. *Dermanyssus* sp. and *Corythucha ciliate* (Figure 1) are such examples of arthropods that occasionally bite humans (Figure 2) [68,69].

## 4. Treatment

Cutaneous symptoms due to arthropods’ bite typically resolve within one or two weeks without intervention. The first-line action in the treatment of arthropods’ bite is cleaning bite spots with soap and water, which prevents secondary skin infection and reduces itchiness. Ice or cold water relieves itching and reduces swelling and inflammation. In the case of persistent itching, the application of topical steroids or oral antihistamines could be beneficial [47]. In the case of a secondary bacterial skin infection, antibiotic ointment or oral antibiotics can be prescribed by physicians. For severe systemic allergic reaction, oral antihistaminic compounds or injection of epinephrine, antihistamine, and corticosteroids are the mainstays of pre-hospital treatment [70,71].

For ticks that remain attached to the skin, tweezers or tick removers will help the removal [72]. Sometimes the rostrum breaks and remains in the skin, without consequence for the bitten person. The rostrum will be rejected later as an external object. In the case of head lice, the louse comb will be helpful for physical removal of the lice and their nits. Medications like ivermectin or chemicals, such as isopropyl myristate, benzyl alcohol, spinosad, dimeticon and malathion are recommended for the treatment of lice infestation [73]. Malathion is also available, formulated as a lotion, shampoo or cream. Laundering (60 °C), dry cleaning, ironing, and replacement of clothing and linens help to remove the body and pubic lice.

## 5. Discussion and Conclusions

**Figure 3:**
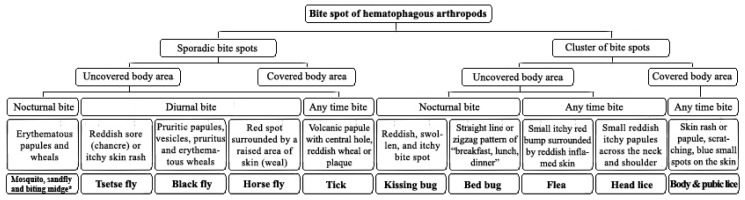
Clinical manifestations of arthropods bite spots. Due to similarities in clinical manifestation and blood feeding activity, the mosquitoes, sandflies and biting midges grouped together.

Physicians and other healthcare personnel are frequently confronted with patients with skin lesions, attributed to the bite of an unidentified arthropod. The reactions include a wide range of clinical manifestations from simple red bumps to allergic or systemic reactions. Without a formal identification of arthropods, the clinical history of the biting together with knowledge of the entomo-epidemiological and clinical signs makes it plausible to infer the putative arthropod species. A flowchart for the discrimination of arthropods’ bites is resumed in the Figure 3. These species-specific criteria help clinical practitioners for precise diagnosis and proposing the proper treatment.

## Figures and Tables

**Table 1:** Entomo-epidemiological criteria of hematophagous arthropods activity.

Hematophagous Arthropod	Vectorial Role		Sex/Bloodfeeding	Stage/ Bloodfeeding	Telmophagy/ Solenophagy	Time/ Bloodfeeding	Seasonal Activity	Geographical Dispersion
	Disease	Pathogenic Agent						
Culicidae (Mosquito)	Malaria, yellow fever, chikungunya, dengue fever, Zika, lymphatic filariasis, and japanese encephalitis	*Plasmodium* sp., Flaviviridae, Togaviridae, *Wuchereria* sp., *Brugia* sp., Japanese encephalitis virus	Female	Adult	S ^1^	Sunset and night *	Commonly warm seasons	Global
Ceratopogonidae (Biting midges) Phlebotominae (Sandflies)	Leishmaniasis, bartonellosis, bluetongue disease, African horse sickness and epizootic hemorrhagic disease, pappataci fever	*Leishmania* sp., *Bartonella* sp., Bluetongue virus, Phlebovirus, Toscana virus, African horse virus, Epizootic hemorrhagic disease virus	Female	Adult	T ^2^	Sunset and night	Commonly warm seasons	Global
Pediculidae, Pthiridae (Lice)	Epidemic typhus, trench fever and louse-borne relapsing fever	*Rickettsia prowazekii*, *Bartonella quintana*, *Borrelia recurrentis*	Both sexes	Nymph and adult	S	Every time	Throughout the year	Global
Ixodidae, Argasidae (Tick)	Lyme disease, Rocky Mountain spotted fever, tularemia, Colorado tick fever, human tick-borne ehrlichiosis, babesiosis, tick paralysis, relapsing fever, Q fever	*Borrelia burgdorferi*, *Rickettsia rickettsii*, *Francisella tularensis*, Colorado tick fever (CTF) virus, *Ehrlichia chaffeensis*, *E. ewingii*, *Babesia microti*, *Borrelia* sp., *Coxiella burnetii*	Both sexes	Larva, Nymph and adult	T	Every time	Throughout the year	Global
Pulicidae (Flea)	Plague, murine typhus, Tungiansis, Tularemia	*Yersinia pestis, Rickettsia typhi*, *Francisella tularensis*	Both sexes	Adult	T	Every time	Throughout the year but mainly inwarm seasons	Global
Glossinidae (Tsetse fly)	Sleep sickness	*Trypanosoma brucei gambiense*, *Trypanosoma brucei rhodesiense*	Both sexes	Adult	T	Diurnal	Throughout the year	Sub-Sahara countries
Reduviidae (Kissing bug, Triatomine bug)	Chagas	*Trypanosoma cruzi*	Both sexes	Nymph and adult	S	Nocturnal	Throughout the year	Latin and South America
Simuliidae (Black fly)	Onchocerciasis (River blindness)	*Onchocerca volvulus*	Female	Adult	T	Diurnal	Throughout the year	Africa, Latin and South America
Tabanidae (Horse fly)	Loiasis	*Loa loa*	Female	Adult	T	Diurnal	Throughout the year	Global
Cimicidae (Bed bug)	-	- **	Both sexes	Nymph and adult	S	Nocturnal	Throughout the year	Global

*: Depends mainly on the species; **: Suspected to be involved in transmission of over 40 pathogens; ^1^: Solenophagy; ^2^: Telmophagy.

**Table 2:** Discriminative clinical characters associated with hematophagous arthropods biting.

Criteria		Hematophagous Arthropod									
		Mosquito	Biting Midge, Sandfly	Louse	Tsetse Fly	Horse Fly	Black Fly	Triatomine Bug	Tick	Flea	Bed Bug
Clinical manifestations		Red itchy papules	Small reddish swollen bump	Small painful red spot or skin rash, inflammation and irritation with blue spots or small spots of blood on the skin	Reddish sore (chancre), itchy skin rash, boil-like swelling	Reddish raised rash	Pruritic papules, vesicules, pruritus and erythematus weals	Red, swollen and itchy skin, anaphylactic shock	Volcanic papule with central hole, reddish wheal or plaque	Small itchy bumps surrounded by reddish inflamed skin, macule, papule, nodule	Macule, papule, nodule, vesicle, bullae, erythematous and pruritic symptoms, allergy, systematic reaction
Bite spot diagnosis	Bite feeling	Painless	Painful	Painful	Painful	Painful	Painful	Painless	Painless	Painful	Painless
	Bite spot pattern	Sporadic and separated	Sporadic and separated	Cluster of separated small red bumps	Sporadic and separated	Sporadic and separated	Sporadic and separated	Cluster of separated small red bumps	separated and sporadic	Cluster of 2, 3 or more bites	Zigzag or straight line
	Biting time	Evening and night	Evening and night	Any time	Diurnal	Diurnal	Diurnal	Night	Any time	Any time	Night
	Location	Exposed area	Exposed area	Throughout body particularly on the scalp, neck and shoulders	Exposed area	Exposed area	Exposed area	Exposed area	Exposed area	Lower extremities, rarely upper body	Exposed area
Treatment		Washing with soap and water, ice compress, antihistamine such as Benadryl, Calamine	Washing with soap and water, ice compress, antihistamine such as Benadryl, Calamine	Combing, pediculicidal shampoo or lotion	Cold compress, calamine, hydrocortisone	Washing with soap and water, ice compress, Benadryl, Calamine	Washing with soap and water, antihistamine such as Benadryl, Calamine	Washing with soap and water, ice compress, epinephrine (in case of systemic reaction)	Removing the tick by tweezers, topical steroids and oral antihistamines	Washing with soap and water, ice compress, Antihistamine such as Benadryl, Calamine	Washing with soap and water, corticosteroids (triamcinolone), antipruritic medications (paroxime, doxepin)
